# A novel strategy sequentially linking mechanical cardiopulmonary resuscitation with extracorporeal cardiopulmonary resuscitation optimizes prognosis of refractory cardiac arrest: an illustrative case series

**DOI:** 10.1186/s40001-022-00711-1

**Published:** 2022-05-28

**Authors:** Linhui Hu, Kaiyi Peng, Xiangwei Huang, Zheng Wang, Yuyu Wu, Hengling Zhu, Jingyao Ma, Chunbo Chen

**Affiliations:** 1 Department of Critical Care Medicine, Maoming People’s Hospital, 101 Weimin Road, Maoming, 525000 Guangdong China; 2 Clinical Research Center, Center of Scientific Research, Maoming People’s Hospital, 101 Weimin Road, Maoming, 525000 Guangdong China; 3 Emergency Department, Maoming People’s Hospital, 101 Weimin Road, Maoming, 525000 Guangdong China; 4 Department of Intensive Care Unit of Cardiac Surgery, Guangdong Cardiovascular Institute, Guangdong Provincial People’s Hospital, Guangdong Academy of Medical Sciences, 96 Dongchuan Road, Guangzhou, 510080 Guangdong China; 5 Department of Critical Care Medicine, Guangdong Provincial People’s Hospital, Guangdong Academy of Medical Sciences, 106 Zhongshan Er Road, Guangzhou, 510080 Guangdong China; 6 The Second School of Clinical Medicine, Southern Medical University, 253 Gongye Dadao Middle, Guangzhou, 510280 China

**Keywords:** Mechanical chest compression, ECPR, Refractory cardiac arrest, Favorable neurological prognosis

## Abstract

**Background:**

Extracorporeal membrane oxygenation (ECMO) to support cardiopulmonary resuscitation (CPR), also known as extracorporeal cardiopulmonary resuscitation (ECPR), has shown encouraging results in refractory cardiac arrest (RCA) resuscitation. However, its therapeutic benefits are linked to instant and uninterrupted chest compression (CC), besides early implementation. Mechanical CC can overcome the shortcomings of conventional manual CC, including fatigue and labor consumption, and ensure adequate blood perfusion. A strategy sequentially linking mechanical CPR with ECPR may earn extra favorable outcomes.

**Case series:**

We present a four-case series with ages ranging from 8 to 94 years who presented with prolonged absences of return of spontaneous circulation (ROSC) after CA associated with acute fulminant myocarditis (AFM) and myocardial infarction (MI). All the cases received VA-ECMO (ROTAFLOW, Maquet) assisted ECPR, with intra-aortic balloon pump (IABP) or continuous renal replacement treatment (CRRT) appended if persistently low mean blood pressure (MAP) or ischemic kidney injury occurred. All patients have successfully weaned off ECMO and the assistant life support devices with complete neurological recovery. Three patients were discharged, except the 94-year-old patient who died of irreversible sepsis 20 days after ECMO weaning-off. These encouraging results will hopefully lead to more consideration of this lifesaving therapy model that sequentially integrates mechanical CPR with ECPR to rescue RCA related to reversible cardiac causes.

**Conclusions:**

This successful case series should lead to more consideration of an integrated lifesaving strategy sequentially linking mechanical cardiopulmonary resuscitation with ECPR, as an extra favorable prognosis of refractory cardiac arrest related to this approach can be achieved.

**Supplementary Information:**

The online version contains supplementary material available at 10.1186/s40001-022-00711-1.

## Introduction

Despite advancements in prevention, intervention, and advanced cardiac life support (ACLS) [1–3], refractory cardiac arrest (RCA), defined by the absence of return of spontaneous circulation (ROSC) after 30 min of appropriate cardiopulmonary resuscitation (CPR), stays a leading cause of mortality globally [4]. Most patients who suffer from RCA do not respond to standard CPR, and outcomes remain poor, with most individuals not surviving [5–8]. In the past decade, extracorporeal life support (ECLS) has shown encouraging results in the resuscitation of RCA irrespective of in-hospital (IHCA) or out-of-hospital (OHCA) [9–13], adult or children cases [14, 15]. Meanwhile, extracorporeal-CPR (ECPR), which adds extracorporeal membrane oxygenation (ECMO) to CPR, is increasingly used in an attempt to improve outcomes [16–20].

When RCA is prolonged, reperfusion of the vital organs by uninterrupted and effective chest compression (CC) before ECMO establishment is the most critical intervention for successful ECPR [21, 22]. However, manual CC decreases in quality over time because of fatigue, impacting ECPR outcomes, especially the neurological prognosis. Mechanical CC devices have the potential to help CPR providers maintain safe, consistent, and high-quality CC in particular settings (e.g., limited rescuers available, prolonged CPR, in a moving ambulance or helicopter, in the angiography suite, during preparation for ECPR, etc.) [23–25]. Even though existing studies [26–28] do not suggest that mechanical CC devices are superior to manual CC when used during resuscitation, little illustrative evidence exists for the effectiveness and safety of sequentially integrating mechanical CPR with ECPR in RCA resuscitation.

## Case series

### Case 1

On July 27, 2021, a 51-year-old male complaining of unrelieved chest distress and pain called emergency medical services. Five minutes after the ambulance's arrival, he suddenly developed CA. His heartbeat was restored but unstable after 10 min of resuscitation on the scene. He was instantly transported to the emergency department (ED), where he continued manifesting ventricular fibrillation (VF) and absence of ROSC for more than 15 min despite the support of mechanical CC by AHS 200B (Physio control), invasive mechanical ventilation (MV), and electric defibrillation. He was then transferred to ICU, where venoarterial ECMO (VA-ECMO) was implemented in about 20 min. After 50 min of ECPR, he reverted to consciousness. Considering his medical history of ischemic heart disease, he was transferred to the interventional radiotherapy room for PCI. Coronary angiography revealed a 90% narrowness of the coronary artery at the proximal anterior descending branch, where a stent was implanted to open clogged vessels. VF was analyzed on ECG monitoring at the end of PCI and transformed to sinus rhythm after the external defibrillator delivered two electric shocks. After sustaining a regular CPR lasting 62 min, followed by an ECPR lasting 190 min, the patient ultimately gained ROSC. Due to prolonged ischemic injury, multi-organ insults involving the heart, lung, liver, kidney, and coagulopathy ensued. Under all means of supportive care by IABP, MV, CRRT, and anti-infective agents, he was weaned off ECMO on Day 6 from admission to ICU and off IABP on Day 11. He was transferred out of ICU on Day 30 and discharged on Day 86 with a cerebral performance category (CPC) of 1.

### Case 2

On July 28, 2021, a 17-year-old girl suddenly collapsed on the street without recording primary diseases. A passer-by, a doctor, immediately identified the CA and started CPR. Further helped by the paramedics dispatched to the site, her heartbeat revived. However, she fell into a coma, manifesting cyanosis, hypopnea of 5 breaths per minute, cardiac acceleration of 130 beats/min, and extreme hypotension hardly to be measured. She was delivered to the ICU in the local hospital for further rescue after an epinephrine injection, where she received intubation and MV to increase oxygen saturation and vasoactive drugs to maintain blood pressure, and finally turned conscious about 2 h later. Unfortunately, about 15 h later, her condition took a sharp turn for the worse, with blood pressure continuing to collapse, leading to a final cardiac arrest. Mechanical CPR started when the absence of ROSC protracted. Our ECPR team was consulted to join the rescue. Instantly, the ECPR team was dispatched about 76 km far away using a transportable ECMO device (ROTAFLOW, Maquet). About 40 min later, the team arrived and established the VA-ECMO support in 33 min. The girl was then transferred to our hospital for further ECPR and extensive monitoring.

Severe metabolic acidosis was detected, with the blood lactic acid higher than 15 mmol/L lingering for 10 h. Meanwhile, methylprednisolone (500 mg iv drip QD over 3 days, followed by 250 mg iv drip QD over 3 days), intravenous immunoglobulin (IVIG, 10 g iv drip QD over 3 days), a high dose of vitamin C (10 g iv drip QD over 1 week), antivirus agents (oseltamivir phosphate capsules 75 mg p.o bid) were medicated for a suspected AFM. Vasoactive drugs (dobutamine, epinephrine, norepinephrine, and levosimendan if indicated and modulated according to blood pressure and myocardial contraction), antibiotics (imipenem, vancomycin, caspofungin, and ceftazidime/avibactam), and nutritional formula (continuous enteral nutritional suspension feeding by a feeding pump at 20 mL/h) were also put on. Due to prolonged low perfusion, she suffered as many as six major organ insults, including brain, heart, lung, liver, kidney, and blood, which necessitated additional support from IABP, CRRT, and MV. After careful brain safeguarding by mild hypothermia therapy and adequate sedation and analgesia, the patient became conscious on Day 2 from ICU admission. After 115 min of mechanical CPR and nearly 44 h of ensued ECPR, she eventually gained spontaneous circulation. Through multidisciplinary discussions, we pursued strict but dynamic protocol on fluid management, blood pressure modulation, and prevention of complications related to ECMO, as detailed in Additional file 1: Table S1. She was weaned off ECMO on Day 7, off IABP on Day 8, transferred out of ICU on Day 21, and discharged on Day 39. We are fulfilled to be acknowledged that she returned to school after hospital discharge.

### Case 3

On November 20, 2021, an 8-year-old girl was transferred from a local hospital to our hospital for carcinogenic shock resulting from suspected AFM. She had a history of runny nose with cough for 2 weeks and syncope for 1 day. Serum troponin I and creatine kinase elevated to 29.79 ng/mL and 2066.61 U/L, respectively. ECG monitor captured ventricular tachycardia rapidly exacerbating to VF. After electric defibrillation, sinus rhythm was restored but unstable. On ICU admission, the girl was lethargic with cold, clammy skin, measuring artery pulse at 54 beats per minute, rapid breathing at 25 beats per minute, and hypotension at 80/51 mmHg with continuous dopamine input through a syringe pump. AFM was clinically diagnosed considering prodromal symptoms of cold accompanied by severe myocardial damage. Methylprednisolone sodium succinate (200 mg iv drip QD over 3 days, halved day-by-day till discontinuation in 3 days), IVIG (0.4 g per kilo of body weight iv drip over 3 days), anti-oxidative (vitamin C 5 g iv drip QD over 1 week) were simultaneously medicated.

Moreover, ECG showed complete atrioventricular block and ventricular escape rhythm, and an ultrasonic cardiogram saw weak cardiac contractions. Cardiogenic shock aggravated despite all intensive care, leading to final asystole. The little girl was intubated and ventilated to start CPR immediately. Unfortunately, stable ROSC was difficult to appear, while continuous CPR largely under mechanical CC lasted more than seventy minutes. The child fell into a deep coma with slowed pupillary light reflex. With her father’s consent, VA-ECMO was established in 30 min to start ECPR. The heart revived to ventricular tachycardia and then sinus rhythm by defibrillation seven hours later. Forty minutes after this ROSC, the girl became conscious again. However, sustaining on-and-off cardiac arrest for 7 h with organs perfused by an advection pressure of about 60 mmHg, she developed a typical ischemic kidney injury. CRRT was introduced to treat oliguria, electrolytic disorder, and acid–base imbalance. ECMO was weaned off on Day 6. On Day 8, for more special children's intensive care, she was transferred to the local Children’s Health Care Hospital, where she got rid of the ventilator and eventually restored her organ’s functionality. She was discharged on Day 32 with a CPC of 1.

### Case 4

On December 30, 2021, a 94-year-old female was admitted to ICU due to breathlessness and unconsciousness. She was clinically diagnosed with pulmonary encephalopathy secondary to acute exacerbation of the chronic obstructive pulmonary disease, which required emergent intubated ventilation. After critical care, she restored consciousness and was later extubated and improved to a generally stable condition which allowed her to be transferred out. On January 14, 2022, the patient suddenly lost consciousness with an ECG monitored VF. She was again intubated, and ventilated by mechanical CC. After 55 min of CPR, the desired ROSC was still absent, whereas the pupils remained responsive to light. Considering her previously relatively fine organ functions, the chance to survive by ECPR still existed. With the consent of the guardians, VA-ECMO support was established. After ECPR started for about 40 min, the ROSC was restored with her upper limbs involuntarily moving. She became utterly conscious two hours later. VF occurred persistently until recurrent electric defibrillation. The patient's condition gradually improved after maintaining electrolyte balance, addressing coagulopathy and anemia, and suppressing infection. No IABP or CRRT was required for the heart rate, and blood pressure tended to be stable. On Day 6, from the initiation of ECPR, ECMO was removed. On Day 14, vasoactive drugs were terminated as blood pressure was normal. On Day 20, she received a tracheotomy to expectorate copious sputum with the expected need for prolonged MV. On Day 26, after the successful removal of ECMO, she died of severe sepsis.

As detailed in Additional file 1: Table S1, we followed a set of general principles and individualized measures when treating the four cases. They consist of removal of underlying causes, brain protection, fluid management, blood control, homeostasis maintenance or other key strategies, which we believe ultimately lead to the desired favorable outcomes. They will be of good help to the medical staff reading this article.

## Discussion

**Fig. 1:**
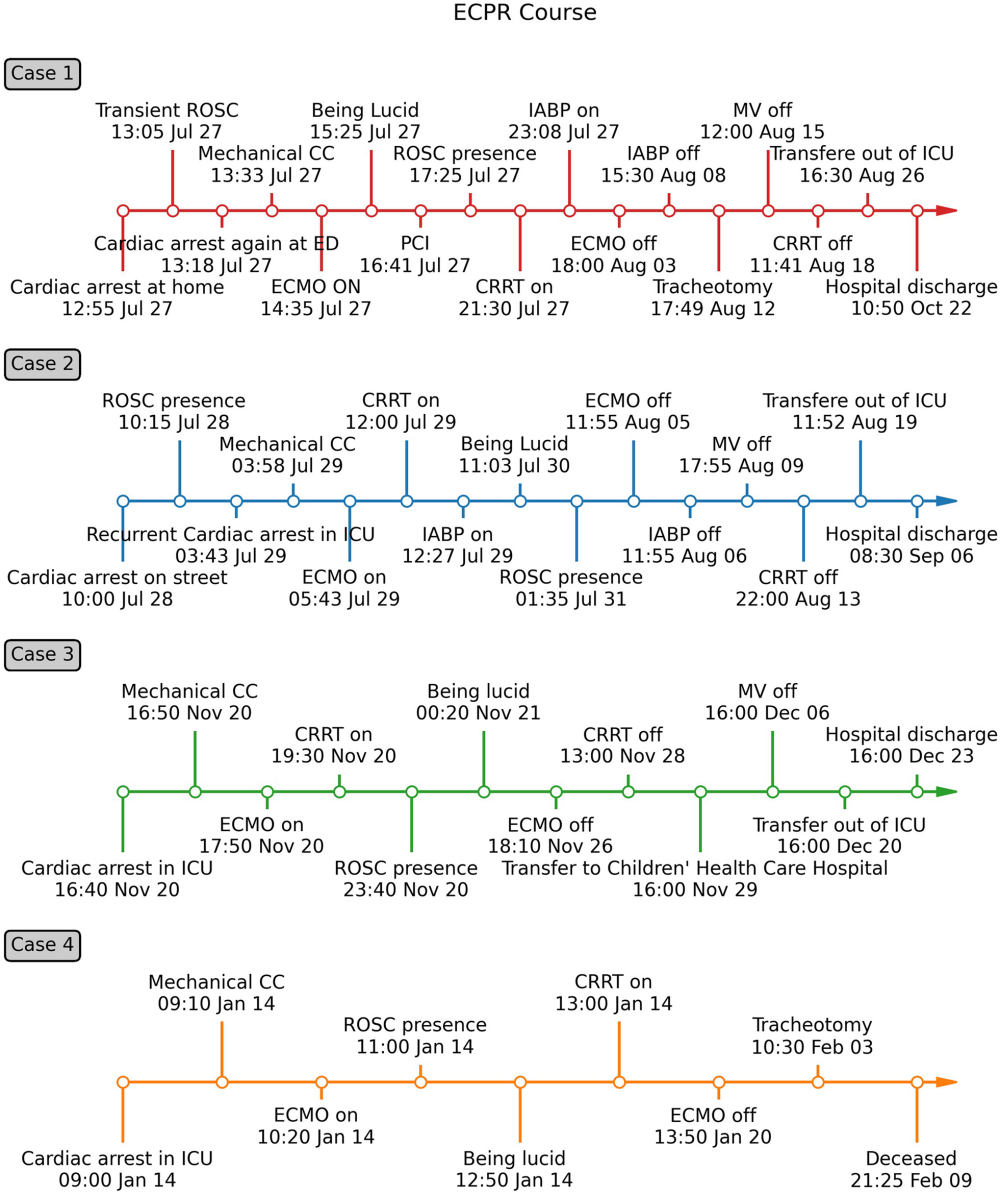
Timeline of main ECPR course for 4 cases with refractory cardiac arrest. Case 1, 2, and 3 received ECPR in 2021, and case 4 in 2022. *CRRT* continuous renal replacement treatment, *ECMO* extracorporeal membrane oxygenation, *ECPR* extracorporeal cardiopulmonary resuscitation, *IABP* intra-aortic balloon pump, *MV* mechanical ventilation, *PCI* percutaneous coronary intervention, *ROSC* return of spontaneous circulation

The four patients included in this ECPR series were diagnosed with refractory cardiac arrest (RCA), with prolonged absences of ROSC crossing from 100 to 2752 min. They had received protracted mechanical CPR before ECMO establishment. All overcame low perfusion, survived the multi-organ failure of the post-cardiac arrest syndrome (PCAS), and eventually recovered to a favorable neurological prognosis. They were discharged from the hospital, except for the very old patient regrettably dying of irreversible sepsis 20 days after successful ECMO removal, as illustrated in Fig. 1. To our best knowledge, this is the first consecutive case series in literature focusing on a new ECPR approach of RCA rescue, involving either myocarditis or myocardial infarction, out-of-hospital or in-hospital, as young as 8 years or as old as 94 years. Our case series highlights a new therapeutic strategy that sequentially integrates mechanical CPR with ECPR, which we can call M-ECPR, for the optimizing management of RCA.

No one should doubt that ECPR has played a vital role in the remarkable success of these 4-case series. ECPR can significantly promote the ROSC in RCA patients, with the rapid initiation of VA-ECMO providing prompt and stable MAP and sufficient oxygenated blood for various organs, especially the brain, heart, liver, and kidney. ECPR reduced cardiac oxygen consumption and preload to rest the damaged myocardium, which broadly enlarged the window of opportunity for ROSC. Meanwhile, controllable target temperature management in resuscitation via ECMO water tank was associated with a more significant reduction in brain injury. It improved the long-term survival and prognosis of the nervous system.

However, the results of ECPR for RCA, traditionally following manual rather than mechanical CPR, are far from satisfactory. It is conventionally believed that short CPR duration was associated with favorable neurological outcomes [29, 30], and widely recognized that ECPR needs to be implemented within 60 min of CA. The CA and ECPR implementation delay was an independent factor in survival [31]. Interestingly, in our case series featuring mechanical CC, a lengthy resuscitation duration before ECPR did not necessarily impact survival and neurological outcome. Therefore, before ECPR starts, mechanical CPR can serve as the prime option for RCA patients’ CPR in various complicated settings of a traffic jam, too long a distance, a shortage of health-care services, etc. Another advantage of this mechanical CPR-bound ECPR strategy is to create equality between patients who live near an ECPR center and others who live further away.

Mechanical CC provides constant, rhythmic, and high-quality CCs that feed blood and oxygen to vital organs. Continuous mechanical CC can reduce medical personnel variance of the compression rhythm and amplitude and fit the heterogeneity of the patient's shape with a fixed device position. Even though many existing studies [26–28] do not suggest mechanical CC devices are superior to manual CC when used in conventional CPR, it is quite another matter when combined with ECPR. A synergistic effect is yielded when sequentially linking mechanical CPR with ECPR, especially in developing regions in China, where CPR resources are relatively limited. Notably, the strategy is not a case of a simple combination of mechanical CPR with ECPR, but a systematic program requiring coordinated alignment and precise management to ensure the final favorable outcome.

First, treatment of reversible underlying causes of RCA is of vital importance, sticking to the American Heart Association CPR guidelines and European Resuscitation Council guidelines [32, 33]. One of the critical roles of ECPR is to bridge the window of opportunity to treat any reversible causes [34]. In Case 1 with suspect AMI, the ECPR preserved blood pressure and oxygenation supply, which produced an opportunity for an emergent PCI to open clogged arteries with stents implanted.

Second, strict management of fluid balance, ECMO flow, and blood pressure plays an essential role in cardio-pulmonary–cerebral resuscitation. On the heart side, the recovery of myocardial function becomes the fundamental problem. At the same time, the reverse flow from VA-ECMO inflated the heart and increased the intraventricular pressure, leading to long-term myocardial injury. Bedside echocardiography helped us perceive the cardiac movement state. When myocardial contraction collapsed during ECPR, the administration of IABP was required to relieve the cardiac afterload and lower the left ventricle pressure of the expanding heart, as the patients of Cases 1 and 2 demonstrated. On the brain side, the association of blood flow and blood pressure with brain perfusion should be individually or personally assessed in light of patients’ primary diseases and age. Prolonged CPR essentially led to brain swelling. We maintained neither excessively low nor overly high cerebral perfusion pressure to avoid aggravating cerebral ischemia or brain edema. Keeping blood oxygen pressure at an adequate but not unduly high level at the early ECPR stage was essential to prevent excessive oxygen from damaging ischemic neurons through oxidative stress.

Third, it is worthy of optimizing the treatment of PCAS, which is mechanistically ascribed to acute systemic ischemia/reperfusion injury after prolonged asystole. All 4 cases suffered multi-organ insults during ECPR, summarized in Table 1. In dealing with the PCAS, multidisciplinary teamwork and various ECLS assistance improved clinical outcomes in CA patients undergoing ECPR [35]. In treating Case 2, who presented a seldom absence of ROSC over 46 h, a team assembled consisting of cardiologists, respiratory physicians, infectious disease experts, and pharmacists. The members argued with counterparts about the optimal treatment regimens at the bedside or online via WeChat. Dynamical medication or invasive interventions were determined and then adjusted through real-time monitoring and analyzing of clinical data reflecting organ injuries, infection, etc. In contrast to the notion by Lazzeri et al. [36], based on our successful clinical practice in this case series, we recommended an early start of CRRT to maintain internal environment homeostasis, which is fundamental to cell and organ recovery. Besides, CRRT can also help remove excess water load accompanied by medication or blood infusion when oliguria, even anuria, occurs following acute kidney injury [37].

**Table 1:** Major organ insults secondary to prolonged ECPR

Measurements	Reference range	Case 1	Case 2	Case 3	Case 4
NT-proBNP, ng/mL	0–300	> 35,000	> 35,000	> 35,000	27,861.3
PCT, ng/mL	< 0.5	12.12	6.76	> 94.42	11.41
cTnI, ng/mL	< 0.15	50	50	41.19	12.22
Lac, mmol/L	0.7–2.5	13.1	> 15.0	> 15.0	> 15.0
Cr, μmol/L	50.4–98.1	577.2	285.6	129.5	124.7
ALT, U/L	5–35	141.6	1491.1	1991.6	45.1
TBIL, μmol/L	3.4–17.1	38.7	72.3	63.1	264.1
PLT, 10^9^/L	100–300	32	21	38	34
Urine output, mL/h	> 17	0.5	0.5	16.3	4.2

Data are shown at the clinically worst level during the ECPR course. All measurements are from blood except urine output

*ALT* alanine aminotransferase, *Cr* creatinine, *cTNI* cardiac troponin I, *Lac* lactic acid, *NT-proBNP* N-terminal pro-brain natriuretic peptide, *PCT* procalcitonin, *PLT* platelet count, *TBIL* total bilirubin

As summarized in Table 2, our case series involves OHCA and IHCA related to AFM or acute MI. ECPR was established in ICU both in a remote hospital and in our hospital. These results are consistent with existing studies [38, 39]. No severe life-threatening complications except lung contusion in Case 2 occurred in the process of automatic CC. The national medical insurance system broadly covered the cost in our case series. In addition to safety and effectiveness, previous analysis [40, 41] proved ECPR to be cost-effective, which laid a good foundation for future extension across China and other developing countries. All these factors increase the generalizability and reliability of the evidence about the efficacy, safety, and cost-effectiveness of this integrated approach.

**Table 2:** Demographic and clinical data

Characteristics	Case 1	Case 2	Case 3	Case 4
Age, years	47	17	8	94
Gender	Male	Female	Female	Female
BMI, kg/m^2^	21.3	18.6	19.7	18.8
Primary diseases	IHD	AFM	AFM	COPD
Site of CA	Home	Street	ICU	ICU
First CPR provider	First-aiders	Passersby	Intensivists	Intensivists
Conventional CPR duration^a^, min	77	120	70	80
Mechanical CC duration, min	62	115	60	70
Cardiac arrest duration^b^, min	270	2752	100	120
Duration for ECMO setup	20	33	30	25
Method for ECMO cannulation	Percutaneous	Percutaneous	Percutaneous	Percutaneous
APACHE II score at ECPR initiation	40	44	22	36
Assistant major intervention(s)	PCI, IABP, CRRT, MV	IABP, CRRT, MV	MV	MV
Mechanical CC-related adverse events	None	Pulmonary contusion	None	None
ECPR-related adverse events	PI, MODS	PE, MODS	PI, MODS	PI, MODS
ECPR duration, day	7	7	6	6
ICU stays, day	30	21	29	33
Hospital stays, day	87	39	20	33
CPC at discharge	1	1	1	Death

*AFM* acute fulminate myocarditis, *APACHE* Acute Physiology and Chronic Health Evaluation, *CA* cardiac arrest, *CC* chest compression, *CPC* cerebral performance category, *CPR* cardiopulmonary resuscitation, *CRRT* continuous renal replacement treatment, *ECMO* extracorporeal membrane oxygenation, *ECPR* extracorporeal cardiopulmonary resuscitation, *IABP* intra-aortic balloon pump, *ICU* intensive care unit, *IHD* ischemic heart disease, *MODS* multiple organ dysfunction syndrome, *MV* mechanical ventilation, *PCI* percutaneous coronary intervention, *PE* pulmonary edema, *PI* pulmonary infection, *ROSC* return of spontaneous circulation

^a^Conventional CPR included manual and mechanical CPR

^b^Cardiac arrest duration, time from cardiac arrest to return of spontaneous circulation

About the transferability of this sequential strategy, some premises are required. The first thing is to establish a quick-response ECMO team capable of being dispatched to the CA scene and establish the ECMO as soon as possible. Our ECPR team consists of trained ICU physicians and nurses with substantial practical experience of over 150 cases of ECMO and several cardiovascular surgeons to backup when surgical cannulation is occasionally required. The time for our team to initiate ECPR averages 25 min. As our team and existing study [42] demonstrated, ECPR implementation by non-surgeons for RCA is also safe and feasible. In a large, international, registry-based cohort study, percutaneous cannulation for VA-ECMO was independently associated with lower in-hospital mortality and fewer complications with a similar risk of severe limb ischemia [43].

**Fig. 2:**
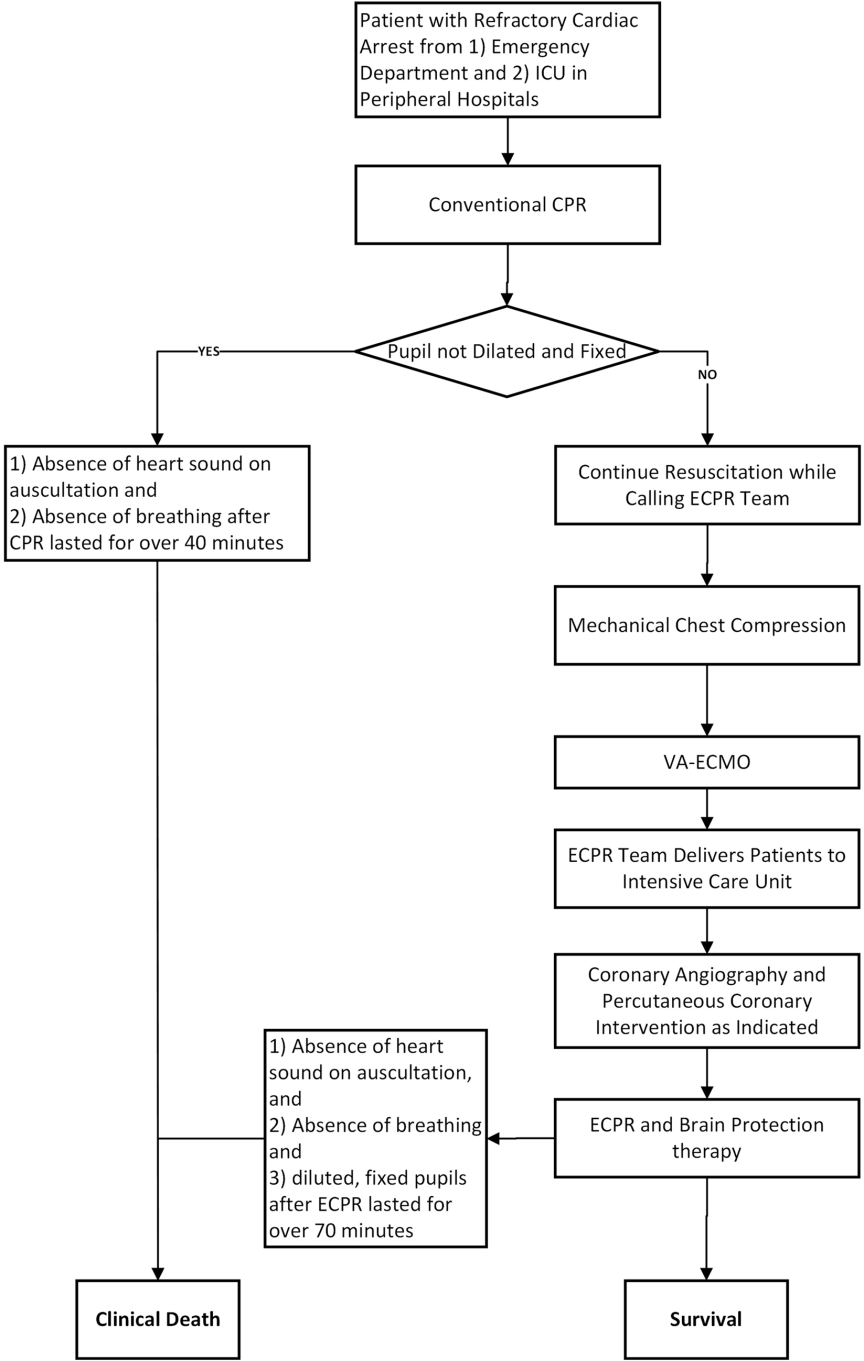
Recommended flowchart of ECPR linked with mechanical chest compression. *ECMO* extracorporeal membrane oxygenation, *ECPR* extracorporeal cardiopulmonary resuscitation, *PCI* percutaneous coronary intervention

Besides, as shown in Fig. 2, a practice-tested and clear-indicated flowchart should be designed to help ECPR team members clarify the implementation target and speed up selecting suitable patients. All 4 cases reached favorable neurological survival despite sustaining a prolonged conventional CPR time ranging from 70 to 120 min. Criteria for application of ECPR will be relaxed regarding CPR time, especially when mechanical CPR was done and the pupillary light reflex existed. We, therefore, referring to the clinical criteria for the determination of death recommended by the World Health Organization [44], decided to play down the CPR time and keep the critical inclusion criteria in the ECPR flowchart as follows: the presence of pupillary light reflex and absence of cerebral hemorrhage, as shown in Fig. 2. Nevertheless, the selection of the patients is a real challenge. A particular scoring system or nomogram may also be prudently used in real-time to select patients with IHCA treated with ECPR to optimize overall outcomes [37, 45].

However, despite the practicality and safety of mechanical CC in RCA, related injuries should be carefully monitored [46]. The long-term outcomes of this ECPR strategy should also be followed up, mainly including health-related quality of life. Further prospective studies, especially randomized control trials with a larger sample size of patients, are still required to confirm the safety and efficacy of this approach.

## Conclusion

Extracorporeal cardiopulmonary resuscitation (ECPR) can be used to support patients with refractory cardiac arrest. This successful case series should lead to more consideration of an integrated lifesaving strategy sequentially linking mechanical cardiopulmonary resuscitation with ECPR as favorable neurological survival can be achieved.

## Supplementary Information

**Additional file 1: Table S1.** General principle and individual treatments during our ECPR.

## Data Availability

The data used or analyzed during the current study are available from the corresponding author upon reasonable request.

## Abbreviations

ACLS: Advanced cardiac life support
AFM: Acute fulminate myocarditis
AMI: Acute myocardial infarction
ALT: Alanine aminotransferase
APACHE: Acute Physiology and Chronic Health Evaluation
APTT: Activated partial thromboplastin time
CA: Cardiac arrest
CC: Chest compression
CPC: Cerebral performance category
CPR: Cardiopulmonary resuscitation
Cr: Creatinine
CRRT: Continuous renal replacement treatment
cTNI: Cardiac troponin I
ECLS: Extracorporeal life support
ECMO: Extracorporeal membrane oxygenation
ECPR: Extracorporeal cardiopulmonary resuscitation
ED: Emergency department
IABP: Intra-aortic balloon pump
ICU: Intensive care unit
IHCA: In-hospital cardiac arrest
IHD: Ischemic heart disease
IVIG: Intravenous immunoglobulin
Lac: Lactic acid
MI: Myocardial infarction
MV: Mechanical ventilation
NT-proBNP: N-terminal pro-brain natriuretic peptide
OHCA: Out-of-hospital cardiac arrest
PCAS: Post-cardiac arrest syndrome
PCI: Percutaneous coronary intervention
PCT: Procalcitonin
PLT: Platelet count
RCA: Refractory cardiac arrest
ROSC: Return of spontaneous circulation
TBIL: Total bilirubin
VA-ECMO: Venoarterial extracorporeal membrane oxygenation
VF: Ventricular fibrillation
