# Supplementary material for: A novel strategy sequentially linking mechanical cardiopulmonary resuscitation with extracorporeal cardiopulmonary resuscitation optimizes prognosis of refractory cardiac arrest: an illustrative case series
Source: Eur J Med Res. 2022 May 28;27:77. doi: 10.1186/s40001-022-00711-1 (PMC9145112; doi:10.1186/s40001-022-00711-1)
Supplement: Supplementary file 1 — Additional file 1: Table S1. General principle and individual treatments during our ECPR. [file 40001_2022_711_MOESM1_ESM.docx]

**Table S1 General principle and individual treatments during our ECPR**

| **General principle** | **Individual treatments** |
| --- | --- |
| Timely treatment of underlying causes | 1. In case of acute myocardial infarction: prompt percutaneous coronary intervention under ECPR support. 2. In case of acute fulminant myocarditis: anti-oxidation (Vitamin C 15 mg/kg, iv drip QD over one week), suppressing myocardial edema (methylprednisolone, 5-10mg/kg iv drip QD over three days, then gradually halve to discontinuation), antiviral agents (oseltamivir, etc.). |
| Brain protection | 1. Mannitol dehydration if the increased intracranial pressure is manifested. Usage: 0.5-1 g/kg iv drip q8h over 3-6 days. 2. Mild hypothermia therapy using ECMO’s thermoregulation system. Maintain the tank temperature at 35 ℃ for 48 hours and later at 37℃. 3. Adequate sedation and analgesia. Maintain early deep sedation with Critical Care Pain Observation Tool (CPOT) at two or lower and Richmond Agitation Sedation Scale (RASS) at -3 or more down, which lasts for 3 to 4 days, to prevent convulsion. Shallow sedation and analgesia are still required later. Commonly used drugs include remifentanil, dextromethorphan, and clomiphene citrate. |
| Blood control | 1. Maintaining target mean artery pressure (MAP). Control MAP at 65 to 70mmHg, ECMO flow at 60 to 80ml/kg, and a relatively normal preload defined by ultrasonic devices at the bedside. 2. Coping with blood advection flows. If the heart does not contract leading to an advection for more than 2 hours, cardiac stimulants (dobutamine, epinephrine, or levosimendan) are administered to maintain a pulsatile blood flow, maintain the essential electrophysiological activity of the myocardium, and prevent intraventricular thrombosis. Balloon pumping is considered when the pulsatile blood flow is hard to gain with heavy cardiac afterload. |
| Stabilization of homeostasis | 1. Dynamical bedside blood gas analysis (BGS). BGS is monitored every 1–2 hours to frame the internal environment status. Homeostasis is maintained through fluid infusion, medication, and blood purification. A negative liquid balance is allowed once the internal environment is relatively stabilized, with the blood lactic acid decreasing. 2. Blood component transfusion to correct anemia, thrombocytopenia, and coagulation disorders. 3. Supplement of albumin. Human albumin injection 10g iv drip q8h for adults and 10g iv drip q12h for the child, both over seven days, to maintain normal albumin levels and alleviate organ edema. |
| Fluid management | 1. Early appropriate relaxation of fluid. For the first 24 to 48 hours of ECPR, the fluid can be appropriately relaxed to keep ECMO smoothly, maintain internal environmental stability, and administer vital medication. 2. Later appropriate restriction of fluid. Negative fluid balance is required when the patient gains a relatively stable condition, which can be judged by indicators as follows: the reduction of vasoactive drugs, the climbing of oxygenation index, the continuous decline of blood lactic acid, and the decrease of central venous-to-arterial partial pressure difference of blood carbon dioxide [P(cv-a)CO_2_] under the same ECMO parameters. |
| Close monitoring of blood coagulation. | The target value for activated clotting time (ACT) is 180–220 seconds, and activated partial thromboplastin time (APTT) is 45–60 seconds. Both of these can be modulated at the lower limit of the target value when bleeding tendency or heartbeat is presented. Unfractionated heparin is used with an initial dosage of 15u/kg, which is adjusted according to the target value to prevent ECMO pipeline or pulmonary membrane thrombopoiesis and obstruction. Protamine neutralization or plasma transfusion is required when ACT or APTT is monitored as excessively high. |
| Airway management | Bedside chest radiographs are reviewed dynamically to detect lung disorders, such as lung atelectasis. The sputum plugs or blood scabs should be removed by broncho-fibro scope to prevent airway obstruction, atelectasis, and bacterial growth. |
| Dynamic multi-disciplinary treatment (MDT) | Establish an instant messaging group (IMG) consisting of nursing staff, and attending, on-duty, and multidisciplinary doctors. The patient’s data will be regularly, or at any time when the dramatic change occurs, fed back in the IMG. The multi-disciplinary doctors will review, discuss the data, and put forward professional consultation to timely modulate the treatment regimen, followed by another round of plan-do-check-action (PDCA) circle. |
| Support and rehabilitation | 1. Immune boosting with intravenous immunoglobulin (IVIG) 0.4 g/kg QD administration. 2. Anti-bacterial. Use broad-spectrum antibiotics early, and adjust antibiotics according to drug sensitivity results and infection indicators such as procalcitonin. 3. Organ functionality protection using blood purification techniques like CRRT. 4. Prevention of stress ulcer bleeding using proton pump inhibitors (PPI). 5. Early enteral nutrition support using pumped-in total nutrient admixture to reduce gastric retention. 6. Daily assessment of the general condition and removing the invasive devices or catheterization as early as possible. 7. Early physical exercise in bed. |

Drug doses are administrated following the package insert if not explicitly mentioned in this Table. g/kg or mg/kg, gram or milligram per kilo of body weight.
